# Lifting the veils on TMEM16E function

**DOI:** 10.1080/19336950.2018.1557470

**Published:** 2019-01-17

**Authors:** Anna Boccaccio, Eleonora Di Zanni, Antonella Gradogna, Joachim Scholz-Starke

**Affiliations:** Institute of Biophysics, Consiglio Nazionale delle Ricerche (CNR), Genova, Italy

**Keywords:** TMEM16, anoctamin, lipid scramblase, gnathodiaphyseal dysplasia, muscular dystrophy, phosphatidylserine

The TMEM16E protein (synonymous to anoctamin 5) has been attracting a great deal of interest, since mutations in the human *TMEM16E* gene were implicated in two different types of hereditary diseases: in gnathodiaphyseal dysplasia (GDD), a rare skeletal syndrome [1], and in muscular dystrophies, limb-girdle muscular dystrophy-2L (LGMD2L) and distal Miyoshi myopathy (MMD3) [2]. Yet for many years, it was not even known which may be the very basic function carried out by this membrane protein, let alone how this function may contribute to physiological and pathophysiological settings. Several facets have contributed to this uncertainty surrounding TMEM16E function. While proteins of the TMEM16 family were initially considered *Ca^2+^-activated chloride channels*, it became clear later that many of them are in reality *Ca^2+^-activated lipid scramblases* mediating the stimulus-induced passive transport of phospholipids, in particular phosphatidylserine (PtdSer), between the leaflets of the membrane bilayer (for review [3]). Moreover, early localization studies indicated that native or heterologously expressed TMEM16E protein was restricted to intracellular membranes and therefore inaccessible to classical approaches like whole-cell patch-clamp and scrambling assays [4].

This uncertainty has now been dispelled. Several studies published in recent years concur on the fact that TMEM16E belongs to the group of family members with Ca^2+^-activated phospholipid scrambling (PLS) activity. A first hint in favour of the “scramblase” option came from a chimeric approach in which a 35-aa-long stretch connecting trans-membrane domains 4 and 5 (designated “scrambling domain” in the TMEM16F sister protein [5]) was swapped between TMEM16E and the plasma membrane-localized TMEM16A. Introduction of the short TMEM16E stretch was sufficient to endow the Ca^2+^-activated chloride channel TMEM16A with lipid scrambling activity [6].

Work from our group provided direct demonstration of Ca^2+^ dependent PLS for the human TMEM16E wild-type protein exploiting its partial plasma membrane (PM) localization following transient overexpression in HEK293 cells [7]. Targeting of a TMEM16E-EGFP fusion to the cell surface was shown by co-localization with the PM marker FM4-64. Additional independent evidence for partial PM localization came from surface biotinylation assays on HEK293 cells stably overexpressing a codon-optimized hTMEM16E version [8]. It is not yet clear if the PM localization of TMEM16E has relevance in its diverse physiological contexts or if it is simply a consequence of protein overexpression. Data from isolated mouse muscle cells indicate that TMEM16E PLS activity may indeed contribute to extracellular PtdSer exposure [8] (see below).

Detection of PLS typically relies on annexin-V binding to PtdSer accumulating in the outer leaflet of the membrane as a consequence of scrambling activity. In both HEK293 cell models [7,8], scrambling assays concurrently revealed annexin-V binding at the cell surface of TMEM16E-expressing cells, which strictly depended on ionophore-stimulated Ca^2+^ entry. Contrarily, HEK293 cells expressing the GDD-related T513I mutant protein showed annexin-V binding independently of Ca^2+^ ionophore application [7]. The most straightforward interpretation of this result is that the T513I exchange causes constitutive TMEM16E scrambling activity at basal cytosolic Ca^2+^ levels, in agreement with a *gain-of-function* phenotype already anticipated from the dominant inheritance mode observed in GDD patients. Moreover, PLS activity at the cell surface of primary muscle progenitor cells derived from a *TMEM16E* knock-out mouse model was reduced compared to wild-type cells [8]. Together, these data strongly suggest that TMEM16E has Ca^2+^-dependent PLS activity.

PLS activity in TMEM16 scramblases is often associated with the development of a non-selective ion conductance, likely originating from the passage of ions along the pathway used for phospholipid transport (for review [3]). TMEM16E makes no exception: membrane currents recorded in transiently transfected HEK293 (and CHO) cells were strongly outward-rectifying, activating at highly depolarized voltages with slow kinetics, and as expected for a Ca^2+^-stimulated protein, the activation threshold shifted towards less depolarized voltages with increasing intracellular [Ca^2+^] [7]. All in all, current properties resembled those of the TMEM16F sister protein [9]. Importantly, current activation of the GDD-related T513I mutant at a given intracellular [Ca^2+^] was significantly shifted towards less depolarized voltages compared to the wild-type protein [7], in agreement with the *gain-of-function* phenotype observed in scrambling assays. By contrast, the membrane currents reported for stably transfected HEK293 cells [8], recorded at the end of an extensive period of PLS activity at high intracellular [Ca^2+^], had essentially linear I-V relationships. It is possible that these recordings may not reflect TMEM16E-mediated ion transport but rather be the consequence of TMEM16E-mediated PLS, leading to an alteration of the membrane structure [7].

Taken together, there is strong evidence in favour of the idea that the TMEM16E protein by itself has Ca^2+^-stimulated scrambling activity: beginning with the swapping experiments using its scrambling domain, the relationship between PLS activity and *TMEM16E* expression in different cellular systems, its similarities to the scramblase TMEM16F, and most importantly, the modification of its Ca^2+^ dependence by a single GDD-related amino acid change. Definitive proof will have to await functional studies with the purified and reconstituted protein. Future work will further have to address the role of TMEM16E-mediated lipid scrambling in bone and muscle tissues.
